# Apparent shift in long-range motion trajectory by local pattern orientation

**DOI:** 10.1038/s41598-017-19005-2

**Published:** 2018-01-15

**Authors:** Ryohei Nakayama, Daisuke Harada, Miyuki G. Kamachi, Isamu Motoyoshi

**Affiliations:** 1 The University of Tokyo, Tokyo, Japan; 2 Kogakuin University, Tokyo, Japan; 3 Present Address: Toppan Printing Co.,Ltd., Tokyo, Japan

## Abstract

The present study shows that the apparent direction of a moving pattern is systematically affected by its orientation. We found that the perceived direction of motion of a single Gabor grating changing position in discrete steps interleaved by blank inter-stimulus interval (ISI) is biased toward the orientation of the grating. This orientation-induced motion shift peaks for grating orientations ~±15 deg away from the physical motion trajectory and was profound for relatively short distances. Orientation adaptation revealed that the directional shift is determined by the apparent –not the physical –orientation of the grating, and a subsequent experiment demonstrated that directional shift is also influenced by the orientation of the contrast-defined stimulus envelope. Results provide further evidence that the apparent trajectory of a motion stimulus is determined by interactions between motion and pattern information at relatively high levels of visual processing.

## Introduction

The mammalian visual system has two distinct pathways –dorsal and ventral visual cortex –that process information about motion/position and shape/color respectively^1,2^. A large body of physiological evidence has shown that most parietal neurons including MT cells are selective for motion regardless of stimulus form whereas cells in inferotemporal cortex and V4 are selective for form and/or color without regards to stimulus motion^3–5^. Such findings are consistent with the notion that, functionally speaking, the two streams have distinct computational goals: the dorsal stream is dedicated to accurate spatiotemporal localization that enables appropriate actions toward external events whereas the ventral stream specializes in recognizing and memorizing objects^6,7^.

In spite of the well-known dichotomy between dorsal vs. ventral brain functions, recent psychophysical studies have revealed robust interactions between stimulus motion and form in the visual system. Perception of local orientation or position is significantly affected by stimulus motion^8,9^ as well as by illusory motion from a static pattern caused by prior motion adaptation^10^. It is also known that motion signals shift the perceived position of a flashed target^11–13^. These findings demonstrate that visual form perception profoundly depends on cortical motion signals^14,15^.

Alternatively, ample evidence shows that motion perception is affected by stimulus orientation. For instance, the detection of a translating light spot is strongly suppressed if the spot moves on a textured background whose orientation is parallel to the spot’s direction of motion^16^. In the suprathreshold regime, the spot’s apparent trajectory is biased in the direction orthogonal to background orientation^17^. Similarly, successive presentations of random dot (Glass) patterns elicit percepts of rotation along their circular organization in the absence of coherent velocity signals^18^. On the basis of these findings, it has been proposed that the visual system integrates local orientation signals with motion signals to estimate motion direction more reliably^19–21^. However, previous psychophysical experiments demonstrating an indirect effect of background stimuli or a global motion percept induced by orientation (cf. Glass pattern^18,22^) likely involve complex factors beyond the integration of motion and orientation.

To shed light on the mechanism of motion-orientation integration, we took the approach of maximal simplicity and studied how the orientation of a single Gabor patch moving on a uniform background affects perceived motion direction^23,24^. Using this display, Experiment 1 demonstrated that the perceived motion trajectory of a Gabor stimulus moving at a specific inter-stimulus interval (ISI) is biased toward the direction parallel to the Gabor’s orientation. Experiment 2 showed that the test Gabor’s trajectory depends on the perceived orientation of an adapted stimulus (tilt aftereffect^25^) rather than on the test’s physical orientation. Experiment 3 revealed that perceived motion direction is affected as much by the orientation/shape of contrast-defined information (i.e., the Gabor’s envelope) as by the orientation of luminance-defined information (i.e., the Gabor’s sinewave carrier). Analysis of the data imply that, in line with other recent investigations^18,26,27^, the perception of motion trajectory is determined by interactions between motion and pattern information at relatively high levels of visual processing.

## Experiment 1

### Method

#### Observers

Two naïve participants and one of authors (DH) participated in Experiments 1–3. Three additional naïve participants and DH participated in the stimulus-position condition of Experiment 1. All participants had normal or collected-to-normal visual acuity. All the experiments were conducted in accordance with the Declaration of Helsinki and were approved by the ethics committee of the University of Tokyo. All participants provided written informed consent.

#### Apparatus

Visual stimuli were generated by a PC (Dell Precision T3400) and presented on a 22-inch CRT monitor (Mitsubishi RDF223H) with a refresh rate of 120 Hz. The spatial resolution of the monitor was 0.039 deg/pixel at a viewing distance of 57 cm. The luminance of each gun was gamma-corrected.

#### Stimuli

**Figure 1:**
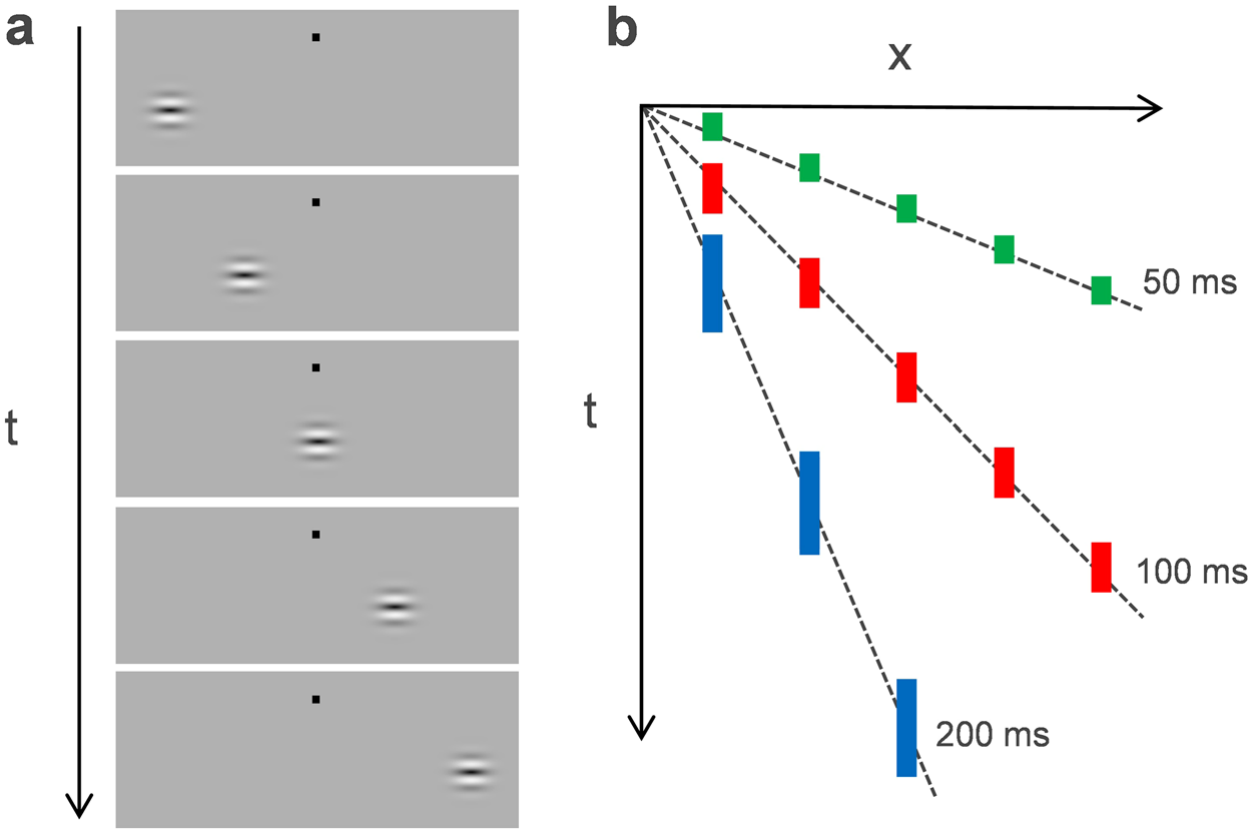
Schematic of the stimulus display and space-time plot of the stimulus presentation used in Experiment 1. (**a**) A Gabor grating is presented successively at different positions every 1.3 deg. (**b**) In one block (as illustrated here), the stimulus appears at five positions with a particular frame duration (50, 100, or 200 ms) and ISI (25, 50, or 100 ms, respectively). In another block, it appears at 5, 9, or 24 positions with a fixed frame duration (50 ms) and ISI (25 ms).

Visual stimuli were composed of Gabor gratings presented in rapid discrete succession on a uniform gray background of W40 × H30 deg (Fig. 1). Gabor stimuli consisted of a sinewave-grating pattern (spatial frequency = 1.6 c/deg, mean luminance = 36 cd/m^2^, luminance contrast = 0.5) tapered by a circular Gaussian window (standard deviation = 0.3 deg). The static Gabor stimulus shifted its position in discrete steps of 1.3 deg between frames, and frames were interleaved by blank frames at variable inter-stimulus intervals (ISI). Therefore, the entire stimulus was perceived as moving from left to right or vice versa. Successive stimulus presentations were positioned along variable diagonal (or horizontal) trajectories. In one experiment block, these presentations were composed of five separate frames (frame duration = 50, 100, or 200 ms) with a blank ISI (25, 50, or 100 ms, respectively). In another block, they were composed of five, nine, or twenty-four frames (frame duration = 50 ms) with a blank ISI of 25 ms. The total presentation time of each trial is determined by sum of the frame duration and ISI duration multiplied by the frame number. Gabor gratings were given various orientations between ±90 deg (0 deg refers to horizontal). Apparent motion trajectory was below a fixation point with a vertical 5.1 deg offset.

#### Procedure

We measured the Gabor’s apparent motion direction by manipulating the trajectory to make it appear as though the Gabor was perceptually moving horizontally (cancellation method). On each trial, observers viewed the stimulus while maintaining central fixation and indicated whether the whole stimulus moved upward or downward (neglecting the trajectory’s horizontal component) by pressing one of two buttons (two-alternative forced choice method; 2AFC). Direction of motion was manipulated according to a staircase method: the diagonal trajectory was shifted downward if observers perceived the Gabor as moving upward in the previous trial and vice versa. The magnitude of vertical increments was determined by pilot experiments. Multiple staircases corresponding to different experimental conditions were run in parallel, and staircases were randomly interleaved across trials. Data were collected for at least 120 trials per condition, and symmetric trials were pooled. For each condition, we defined the point of subjective equality (PSE) as the direction of motion corresponding to 50% (i.e., chance reporting) calculated by the maximum likelihood method^28–30^. To express data in terms of perceived direction, we simply reversed the sign of the Gabor’s physical direction. Standard error was estimated via the bootstrap method (1000 samples)^31–34^.

## Results

**Figure 2:**
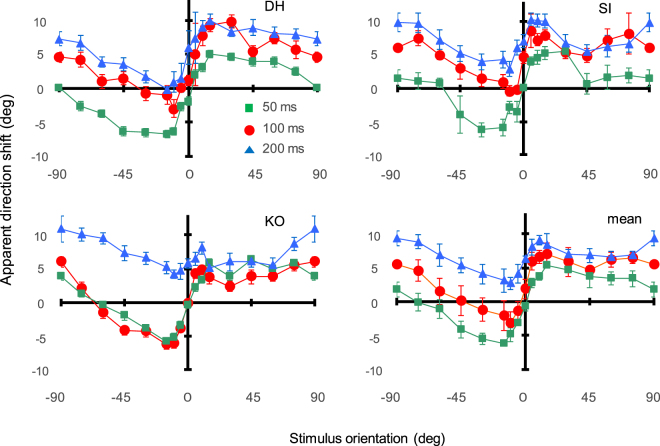
Apparent motion direction as a function of Gabor orientation. Green, red, and blue circles show results for frame durations of 50, 100, and 200 ms. Panels shows results for each observer as well as cross-observer averages (bottom right). Error bars represent ± 1 s.e.m.

Figure 2 shows apparent motion direction as a function of Gabor orientation. Each curve shows the results for different frame durations. For all observers, apparent motion direction was shifted systematically towards stimulus orientation. The perceived shift in direction increases as frame duration decreases. Note that apparent motion direction tended not to be horizontal even if stimulus orientation was horizontal (0 deg) typically for the frame duration of 200 ms [t-test: *p* = 0.057 across frame durations; *p* = 0.004 for 200 ms]; instead, we found that perceived direction is biased toward the direction pointing away from the fixation point. This centrifugal bias can be accounted for by the visual system’s intrinsic preference for motion moving away from the fovea^35,36^.

**Figure 3:**
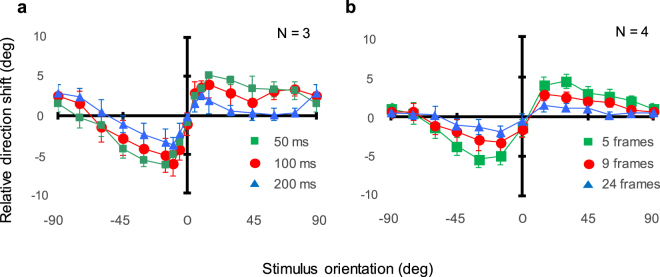
Apparent motion direction as a function of Gabor orientation with centrifugal bias subtracted. (**a**) Results for the frame-duration conditions averaged across observers. Green, red, and blue circles show results for frame durations of 50, 100, and 200 ms. (**b**) Results for the frame-number conditions averaged across observers. Green, red, and blue circles show results for five, nine, and twenty-four frames. Error bars represent ± 1 s.e.m.

We compensated for centrifugal bias by subtracting the average shift of the apparent motion direction across orientations from the data for each observer and stimulus condition. Figure 3 shows the compensated data averaged across observers. Panels show results for three different frame durations (a) and numbers (b). For the frame-duration conditions, a two-way ANOVA reveals the main effect of the orientation [ANOVA: *F*_(16,32)_ = 10.717, *p* < 0.0001] (but not of the frame duration [ANOVA: *F*_(2,4)_ = 4.644, *p* = 0.091, *n.s*.]) along with the interaction between orientation and frame duration [ANOVA: *F*_(32,64)_ = 1.969, *p* = 0.011] in units of the relative shift into the stimulus orientation. The relative shift at −15 deg exceeds the shifts at −90–45 deg [*p* < 0.02] and 0 deg [*p* < 0.0001] while the relative shift at 15 deg is also larger than those at 0 deg [*p* < 0.0001] and 45 deg [*p* = 0.047]. Similarly, for the frame-number conditions, a two-way ANOVA reveals a main effect of the frame number [ANOVA: *F*_(2,6)_ = 17.686, *p* = 0.0031] and orientation [ANOVA: *F*_(12,36)_ = 12.647, *p* < 0.0001] along with an interaction between them [ANOVA: *F*_(24,72)_ = 5.096, *p* < 0.0001]. The relative shift at −15 deg exceeds the shifts at −90–60 deg [*p* < 0.0002] and 0 deg [*p* < 0.0001] while the relative shift at 15 deg is larger than those at 0 deg [*p* < 0.0001] and 75–90 deg [*p* < 0.01]. All curves suggest that the relative shift peaks, or becomes saturated, at a Gabor orientation of about ± 15 deg and that the magnitude of the shift is about 5–6 deg at most in the direction parallel to stimulus orientation.

**Figure 4:**
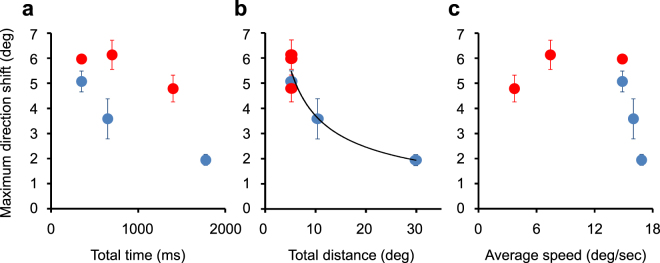
Effect of spatiotemporal parameters on maximum orientation-induced motion shift. (**a**) Maximum direction shift is plotted as a function of total presentation time (**a**), distance (**b**), and average speed (**c**). Red circles show results for frame-duration conditions and blue circles show results for frame-number conditions. The solid curve is a power function fitted to the data in (**b**). Error bars represent ± 1 s.e.m.

To quantify the magnitude of the shift, we averaged the absolute maximum shift in each stimulus condition across observers. The maximum shift decreases with frame number [ANOVA: *F*_(2,6)_ = 25.439, *p* = 0.001] but not with frame duration [ANOVA: *F*_(2,4)_ = 6.297, *p* = 0.058, *n.s*.]. Figure 4 plots maximum shift as a function of total time (Fig. 4a), distance (Fig. 4b), and average speed (Fig. 4c) of successive stimulus presentations. Red and blue circles are maximum shifts for the frame-duration and frame-number conditions respectively. Maximum shift does not show a strong correlation with total presentation time (Fig. 4a) while it decreases monotonically as total distance increases and a power function fits the data very well (Fig. 4b; y = 14.472 ×^−0.592^; R^2^ = 0.95). One cannot dissociate the effects of frame duration and ISI since they covaried and, presumably, shift amount may have relevance to the subjective strength of apparent motion such as poorer motion with longer ISIs and better motion with longer motion extents^37^. As a function of speed, maximum shift is greatest for speeds up to about 15 deg/sec and then decreases dramatically (Fig. 4c). The apparent shift may occur only for slower speeds, but it is difficult to reason the sharp decline. These analyses imply that apparent motion direction is affected by local Gabor orientation and that the effect largely depends on frame number, that is, total presentation distance.

## Experiment 2

The results of Experiment 1 indicate that orientation signals, which are presumably coded by orientation-selective neural channels^38,39^, induce shifts in perceived motion trajectory. However, another possibility still remains. That is, the dynamic presentation of grating stimuli may directly distort the sensory input involved in computing motion direction. To examine this possibility, we combined the experimental manipulations of Experiment 1 with an adaptation paradigm designed to induce a tilt aftereffect (TAE) –after adapting to a slightly tilted Gabor grating, a physically vertical test Gabor is perceived as tilted away from the adaptor^25^. This illusory tilt is generally thought of as resulting from neural processing of orientation signals in visual cortex. If the orientation-induced motion shift depends on the orientation signals coded in cortex, then the motion shift of a Gabor test should also depend on the apparent orientation induced by the adaptor.

## Method

**Figure 5:**
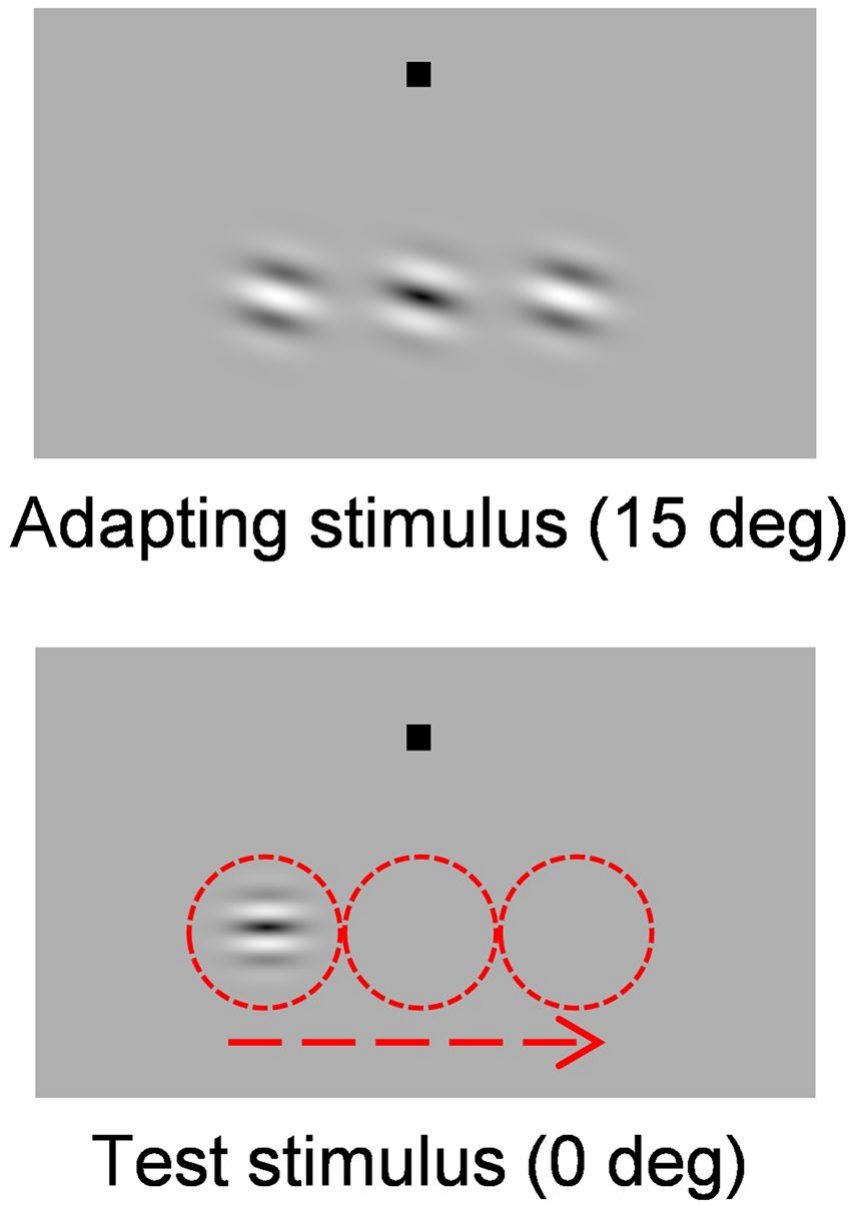
Schematic of the adaptor and test stimulus used in Experiment 2. Red circles indicate the positions where a Gabor grating is presented successively (circles were not presented in the actual experiments).

As in Experiment 1, the test stimulus consisted of a Gabor grating with the exception that Gabor orientation was fixed to 0 deg (horizontal). The test Gabor was sequentially presented at three different positions separated by 1.3 deg (frame duration = 100 ms; blank ISI = 50 ms). The adaptor stimulus consisted of three Gabor gratings oriented +15 deg (or −15 deg) away from horizontal (to maximize the tilt after-effect) presented simultaneously (Fig. 5). Each of the three adaptor gratings drifted within their envelope and reversed direction according to a triangular wave at a temporal frequency of 1.25 Hz (i.e. slow smooth motion)^40,41^, thereby preventing the formation of afterimages and/or a tilt aftereffect locked to the same phases between adaptor and test gratings. The adaptors were positioned horizontally and parallel to each other and where presented for 30 sec before each experimental block and represented for 4 sec at the beginning of each trial. The test stimulus appeared 500 ms after the adaptation period, and top-up adaptors were positioned at the same location as the test stimulus to maximize the adaptation aftereffect. We measured apparent motion direction with and without adaptation using the same procedure as in Experiment 1.

## Results

**Figure 6:**
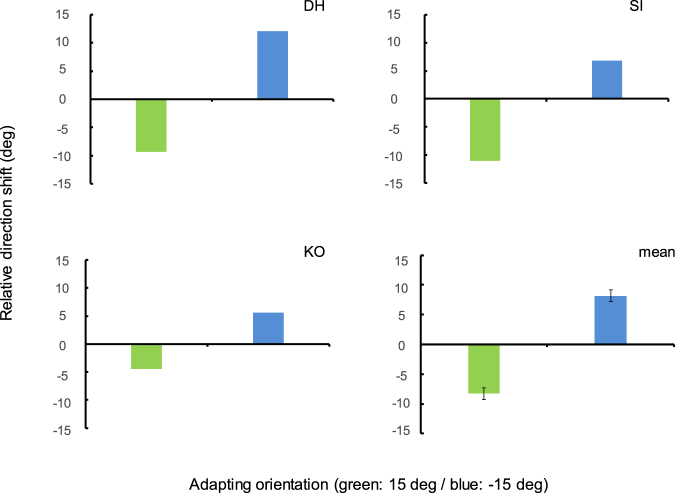
Effect of apparent orientation on the orientation-induced motion shift. Bars show the *difference* in the magnitude of directional shift between adapted and non-adapted conditions. Colored bars represent adaptor gratings tilted by ±15 deg (green bars = +15 deg; blue bars = −15 deg). Panels show results for each observer and for the cross-observer average (bottom right). Error bars represent ± 1 s.e.m.

Figure 6 shows the *difference* in the magnitude of the apparent-motion direction shift between adapted and non-adapted conditions. Colored bars show results for different adaptor orientations (green bar =  + 15 deg; blue bar = −15 deg). Given that the physical orientation of the test stimulus was horizontal in both the adapted and non-adapted conditions, one would expect no differences in apparent motion direction between conditions if direction depended on the orientation of the test or relevant stimulus parameters. In actuality, however, apparent motion direction is shifted toward the same direction as the apparent orientation induced by the adaptor [t-test: *p* = 0.039]. These results suggest that the orientation-induced shift in motion direction depends on the *apparent* orientation of the test Gabor, not on its physical orientation.

## Experiment 3

In Experiments 1 and 2, the orientation-induced motion shift was observed for luminance-defined gratings, but it remains unclear whether the apparent shift in motion direction is produced only by the processing of luminance orientation signals in the early stage of vision or, additionally, by the processing of second-order orientation signals in higher stages of pattern processing. In Experiment 3, we examined if apparent shifts in motion direction occur for test stimuli whose second-order Gaussian window was oriented.

## Method

**Figure 7:**
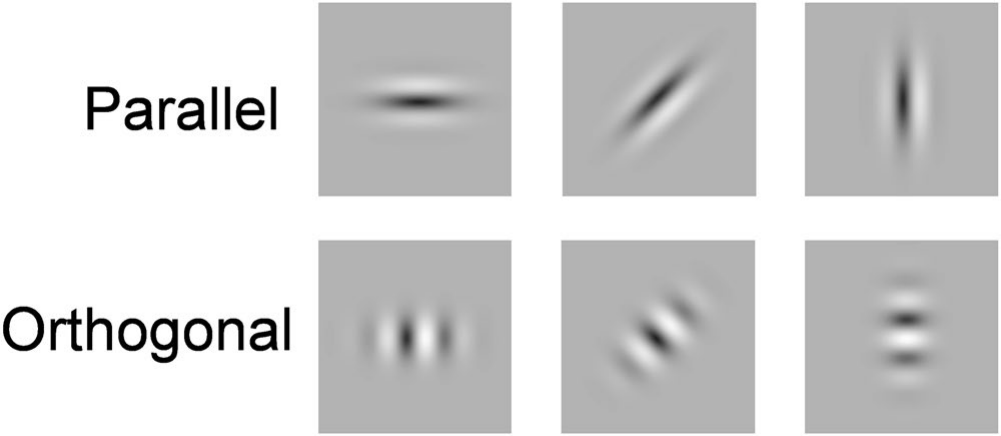
Visual stimuli used in Experiment 3. The orientation of the grating pattern was either parallel (top) or orthogonal (bottom) to the major axis of the elliptical Gaussian window.

Test stimuli consisted of a sinusoidal grating tapered by an elliptical Gaussian window (Fig. 7). The major and minor axis of the elliptical window was 0.6 and 0.3 deg in standard deviation, and the orientation of the grating was either parallel or orthogonal to the major axis of the elliptical window. As before, test stimuli were presented at five successive positions separated by 1.3 deg (frame duration = 50 ms; blank ISI = 25 ms). We measured the apparent-motion direction shift as a function of the window’s major axis orientation. Other configurations and procedures were same as Experiment 1.

### Data availability

The dataset is available online on figshare public repository.

## Results

**Figure 8:**
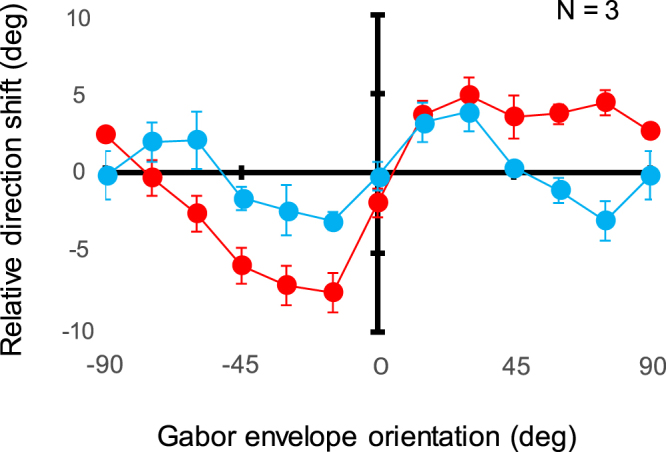
Apparent motion direction as a function of the orientation of the elliptical stimulus window. Data were compensated for centrifugal bias and averaged across observers. Circles show results for gratings either parallel (red) or orthogonal (blue) to window orientation. Error bars represent ± 1 s.e.m.

Figure 8 plots apparent motion direction (compensated for centrifugal bias) as a function of window orientation. A two-way ANOVA reveals the main effect of window orientation [ANOVA: *F*_(1,2)_ = 20.499, *p* = 0.046] and grating orientation [ANOVA: *F*_(12,24)_ = 20.674, *p* < 0.0001], and the interaction between them [ANOVA: *F*_(12,24)_ = 7.119, *p* < 0.0001] in units of the relative shift in stimulus-window orientation. The relative shift at −15 deg exceeds the shifts at −90–45 deg [*p* < 0.04] and 0 deg [*p* < 0.0001] while the relative shift at 15 deg is larger than those at 0 deg [*p* < 0.0001] and 45–90 deg [*p* < 0.05]. These analyses indicate that relative shift peaks around ±15 deg and is larger in the parallel condition compared than in the orthogonal condition. In the orthogonal condition, relative shift at −75 deg is below shifts at −90 deg [interaction: *p* = 0.045] and −45 deg [interaction: *p* = 0.001] while relative shift at 75 deg is smaller than shifts at 90 deg [interaction: *p* = 0.008] and 45 deg [interaction: *p* = 0.02]. Thus, relative shift bottoms out around ±75 deg and, especially, below zero at ±75 deg [t-test: *p* = 0.031]. In cases where grating and window were parallel (red circles), apparent motion trajectory is shifted towards the direction common to both the window and the grating. Results were somewhat complicated in cases where the grating was orthogonal to the window (blue circles). For orientations around ±15 deg, the perceived direction shift is towards the orientation of the window’s *major* axis; for orientations around ±75 deg, apparent direction is shifted towards the window’s *minor* axis. These results suggest that apparent motion direction is affected not only by the orientation of the luminance grating but also by the orientation of the contrast-defined window. Furthermore, the illusory directional shift seems to be attracted either to grating orientation or to window orientations closer to horizontal.

## Discussion

The present study has demonstrated that the orientation of a single moving Gabor pattern markedly affect its perceived direction of motion. This illusory shift in motion direction increases if duration is short and the presentation only lasts a few frames (Experiment 1). Perceived directional shifts also depend on *apparent* stimulus orientation as induced by the tilt aftereffect –a bias in perceived orientation triggered by prolonged viewing of an off-orientation adaptor that presumably alters the balance of repulsive interactions between orientation-selective channels –rather than on physical stimulus orientation. On this basis, the illusory shift is likely related to orientation signals coded in the visual system (Experiment 2). Experiment 3 revealed that the shape of the whole stimulus (i.e., the orientation of the Gabor’s window) produces shifts in perceived motion direction as large as shifts induced by the orientation of the luminance-defined patterns.

Recently, Hughes *et al*.^42^ had observers localize apparent position as extrapolated from the motion trajectory of grating stimuli and revealed that directional shifts in motion trajectory depend on the grating orientation. The directional shift found in our study (~6 deg) is much larger than that reported in the Hughes *et al*. study (~1.80 deg). One potential explanation for this discrepancy is the difference in spatial resolution between foveal and peripheral vision –our stimuli were viewed peripherally (5.1 deg) whereas Hughes *et al*. allowed observers to track the moving stimulus and localize it foveally. In this respect, results from Hughes *et al*. could be interpreted as evidence that directional shifts depend not only on retinal inputs but also on head/body-centered stimulus motion that activates mechanisms that specifically process motion in non-retinal coordinates. Secondly, the fact that Hughes *et al*.^42^ employed continuously-moving stimuli may have weakened the effects relative to those in the present study. It is widely known that continuous (or “short-range”) motion is detected by early motion energy detectors whereas long-range motion (produced by our stimuli with discrete-jumps and blank ISIs) is processed by higher-order motion detectors^43,44^. Together, Hughes *et al*.’s results and ours provide joint evidence that orientation information is referenced in higher-order motion processing stages and causes illusory shifts in motion direction. This conclusion is consistent with psychophysical findings that: 1 - the effect of orientation on motion direction takes place at processing stages subsequent to the integration of motion over multiple local apertures^26^, 2 - the effect persists even if orientation information is presented spatially separately from the moving stimuli^45^, and 3 - global motion perception depends on local orientation informastion^18,22,27^.

Results of Experiment 3 suggest that motion shift depends on the shape orientation of contrast-defined stimulus windows that would involve higher-order orientation processing. Seminal studies on so-called motion “streaks” have established intimate links between perceived motion direction and orientation signals encoded in low-level visual cortex such as area V1^16,19,20,46^. Dakin, Williams, & Hess^47^ studied the combined effects of luminance (grating) and contrast (window) of a stationary stimulus on its apparent orientation, and their findings lead us to suggest an alternative interpretation of our results. Indeed, the shift in motion direction might be indirectly due to the distortion of luminance orientation information by the orientation of the contrast window. However, physiological and psychophysical evidence has shown that second-order orientation information is directly processed at higher stages of visual processing including V2^48^ and influences percepts^49,50^. In agreement with these findings, orientation information responsible for the illusory shift of motion direction would consist of higher-order contour/shape orientation along with first-order orientation energy produced at V1. According to this view, in Experiment 2, adaptation to physical orientation might have affected motion direction as a consequence of a shift in the orientation represented at higher levels. Given that the effect of adaptation to an orientation on apparent motion is much broader than on orientation itself ^51^, the high-level representation of orientation may affect apparent motion and orientation in a distinct and independent manner.

In order to solve the aperture problem and compute velocity, higher-order motion processing must integrate motion information over different locations and over a wide range of directions and spatial frequencies^52,53^. However, motion direction is not necessarily determined immediately and exclusively by integrating motion information. It is sensible that orientation information should also assist higher-order motion processing if it can contribute to more accurate estimates of motion direction. The orientation-induced shift in perceived motion direction is increased by shorter motion trajectories as well as by faster motion (<15 deg/sec)^42^. Note that we found a subtle proportional increment of the maximum shift when apparent motion became faster up to about 15 deg/sec (Experiment 1) and speed will be another factor of the directional shift or even it might be a by-product of speed modulated by orientation information^54^. These trends suggest that high-level motion mechanisms may weigh the integration process in favor of orientation information relative to motion information if low-level motion signals are ambiguous at stimulus onset. This conforms to other findings that accurate direction perception largely depends on the strength of motion signals such as stimulus contrast and path length^52^. Indeed, the human perceptual system appears to rest on a common principle that solutions to ambiguous inputs from a particular module are further constrained by using reliable information from other sensory inputs, as exemplified by interactions between sensory modalities (e.g. ventriloquism effects^55,56^; cross-modal Bayesian estimate^57^).

In conditions where the grating is oriented perpendicularly to the shape orientation of stimulus windows, Experiment 3 has shown that the illusory shift in motion direction is based either on grating orientation or on shapes whose orientation are closer to horizontal. It is generally known that oblong stimuli presented parallel to the horizontal axis are perceived more finely and that oblique orientations are susceptible to many illusory phenomena. The oblique effect is also true of orientation-induced motion shifts that become larger for diagonal motion but remain unobserved for horizontal motion of tilted line stimuli^26,58^. Importantly, the present study exhibits direction shifts for horizontal motion trajectories, but this is not surprising given that, as mentioned above, grating stimuli provide more reliable orientation information relative to line stimuli whereas apparent motion provides more ambiguous motion information relative to continuous motion. These findings suggest that magnitude of the directional shift could result from the summation of two sources of orientation information –luminance-grating orientation and contrast-window orientation – that are strongest at orientations close to horizontal and weakest at non-horizontal orientations. Accordingly, the illusory shift is smaller in the orthogonal condition relative to the parallel condition and disappears if stimulus orientation is around ±45 deg in the orthogonal condition as two opposite effects of equal magnitude cancel each other out (Fig. 8).

A quantitative analysis of the present data leads us to consider a simple model of interactions between orientation and motion information. It could be achieved by fitting a couple of amplitude reversed orientation filters to luminance- and contrast-defined orientations^59^: the negative one centered at about −15 deg; the positive one peaking around +15 deg. This is consistent with the fact that the tilt aftereffect is maximized by adapting to the first- or the second-order orientation around ±15 deg^49^. Alternatively, the difference between the parallel and orthogonal conditions in Experiment 3 could be interpreted in terms of luminance orientation energy that, computationally, should become at most 1.5 times higher for parallel stimuli than for orthogonal stimuli depending on the size of the applied Gabor filter. However, taking into account that the maximum shift in the parallel condition is 2.0 times lager compared to the orthogonal condition, both levels of orientation energies (luminance and contour/shape) would involve the direction shift of apparent motion in a quasi-additive manner.
